# Smart Cardiac Framework for an Early Detection of Cardiac Arrest Condition and Risk

**DOI:** 10.3389/fpubh.2021.762303

**Published:** 2021-10-22

**Authors:** Apeksha Shah, Swati Ahirrao, Sharnil Pandya, Ketan Kotecha, Suresh Rathod

**Affiliations:** ^1^Computer Science Department, Symbiosis Institute of Technology, Symbiosis International (Deemed University), Pune, India; ^2^Symbiosis Centre for Applied Artificial Intelligence, Symbiosis Institute of Technology, Symbiosis International (Deemed University), Pune, India

**Keywords:** artificial intelligence, cardiac arrest prediction, machine learning, predictive analysis, risk classification, heart failure

## Abstract

Cardiovascular disease (CVD) is considered to be one of the most epidemic diseases in the world today. Predicting CVDs, such as cardiac arrest, is a difficult task in the area of healthcare. The healthcare industry has a vast collection of datasets for analysis and prediction purposes. Somehow, the predictions made on these publicly available datasets may be erroneous. To make the prediction accurate, real-time data need to be collected. This study collected real-time data using sensors and stored it on a cloud computing platform, such as Google Firebase. The acquired data is then classified using six machine-learning algorithms: Artificial Neural Network (ANN), Random Forest Classifier (RFC), Gradient Boost Extreme Gradient Boosting (XGBoost) classifier, Support Vector Machine (SVM), Naïve Bayes (NB), and Decision Tree (DT). Furthermore, we have presented two novel gender-based risk classification and age-wise risk classification approach in the undertaken study. The presented approaches have used Kaplan-Meier and Cox regression survival analysis methodologies for risk detection and classification. The presented approaches also assist health experts in identifying the risk probability risk and the 10-year risk score prediction. The proposed system is an economical alternative to the existing system due to its low cost. The outcome obtained shows an enhanced level of performance with an overall accuracy of 98% using DT on our collected dataset for cardiac risk prediction. We also introduced two risk classification models for gender- and age-wise people to detect their survival probability. The outcome of the proposed model shows accurate probability in both classes.

## Introduction

At present times, cardiovascular disease (CVD) is one of the most contagious illnesses. According to WHO, ~60% of cardiac patients are Indians to suffer from CVDs. Cardiac symptoms are generally associated with dynamic changes in an individual. The vital changes in the human body may lead to cardiac problems due to misdiagnoses or improper treatment. Another sign of cardiac disease is environmental changes and lifestyles of people (1). In healthcare, CVDs are considered a vital aspect to be diagnosed as soon as possible to minimize the risk. Hence, early prediction of CVDs needs to be examined in a patient (2, 3).

Cardiovascular disease comprises heart or cardiac diseases in heart patients and is a critical challenge in the medical field. Several reasons and findings that cause cardiac arrest are observed, such as a change in personal and professional lifestyles, habits, inactive lifestyle, growing age, habitual history related to smoking, alcohol consumption, stress level, and physiological signs, such as diabetes, high level of blood pressure (BP), obesity, cholesterol, hypertension, and existing heart problems. These risk factors need early, accurate, and efficient diagnosis to prevent cardiac arrests (4–6). The risk factors can be depreciated by executing proper lifestyle activities, such as lowering salt usage, absorbing a healthy diet, prohibiting alcohol and tobacco use, and regular physical exercise (7). Medical sectors use the abovementioned vital signs to produce consequential information from data. The vital signs are generally collected from publicly available datasets, wearable gadgets, medical hospitals, or sensors. Monitoring health parameters using Internet-of-Things (IoT) is a forming trend for future well-being. IoT sensors are mostly used to collect real-time vital signs and monitor the health parameters of individuals (8). Collecting, processing, and analyzing vitals help predict risk early to tackle the problem (9, 10).

One of the broad areas of Artificial Intelligence (AI) is machine learning (ML). ML is an effective technology and efficient field, which is based on prediction purposes. Using ML concepts, we can develop models or enable human abilities and train our data collected or gathered from the past for future predictive analysis (11). Those data are further divided into training and testing, which help in predicting future possibilities. This combined technology is called ML (12). The ML algorithm needs pertinent information for training and testing. In addition, the model performance can be raised if the balanced data are given to the ML model. In addition to that, the capability and accuracy of the predictive model can be improved if relevant features are selected from a dataset. Hence, a balanced dataset and feature selection are paramount for improving model performance (13). Furthermore, for cardiac arrest prediction, the ML algorithm is used, which has been addressed in this research. Nowadays, deep learning models show prominent growth and improvement in predicting and analyzing heart diseases (14).

Several studies have shown predictions on publicly available datasets. Still, this research aims to find early risk prediction of cardiac arrest based on real-time data collected from an individual using sensors and equipment. Furthermore, the authors build a Neural Network, Bagging, and Boosting model using ML techniques by selecting relevant datasets to show which model accomplishes best. This study also aims to classify risk in different age and gender groups of people. The points mentioned below show the main contributions of the article:

- To collect real-time vital signs of an individual using sensors and equipment.
- To detect cardiac arrest by applying the ML algorithm.
- To show a comparative analysis of the various ML classifiers.
- To classify cardiac arrest risk for the coming 10 years in different age groups.
- To show survival probability gender-wise in our creation of data.

The rest of the article is assembled as follows. Section Related Work narrates research work on heart diseases along with existing methods, datasets, and techniques available. Section Proposed Work exhibits the study of the proposed study of the author. Section Methodology reveals the methodologies and algorithms used for disease risk prediction. Section Risk Classification conveys the risk classification in people. Section Exploratory Data Analysis presents the results of Exploratory Data Analysis. Section Implementation Details communicates the implementation details of all the experiments and results. Finally, section Discussion, Conclusion, and Future Work discusses and concludes the research study of the author.

## Related Study

### Literature Survey

This section discusses the existing article to predict and diagnose heart diseases using several techniques and datasets used with various features and classification techniques.

Ambekar and Phalnikar (1) proposed a risk model for heart disease with structured data using a Convolutional Neural Network-based Unimodal Disease Risk Prediction (CNN-UDRP) model to classify risk levels as high, low, and medium. The dataset was extracted from the UCI repository. To avoid missing values, performed data cleaning and imputation to substitute missing values and increase the performance of the model were implemented. The proposed model achieves an accuracy of nearly about 65%. Ramprakash et al. (2) developed a model using a Deep Neural Network (DNN) and χ^2^–a statistical method to predict the risk of heart patients. The Cleveland dataset was used, which is publicly available on the UCI repository. In this study, DNN models Artificial Neural Network (ANN) and DNN and proposed statistical methods χ^2^-ANN and χ^2^-DNN were compared. The comparison shows that the proposed method χ^2^-DNN was more efficient in providing an accurate accuracy of 94%. Maini et al. (5) discuss data mining techniques to detect heart disease risk at an early stage and show the importance of assigning a cloud-based approach for helping healthcare providers. Mohan et al. (6) proposed a hybrid model combination of Linear model and Random Forest (RF) technique with different features combined on publicly available datasets, i.e., Cleveland from UCI repository and compared its result with models of Naïve Bayes (NB), RF, Linear model, Deep Learning, Logistic regression, etc. From all the proposed models, Random Forest with Linear Model (HRFLM) outperforms all others with 88.4% accuracy. Shah et al. (3) used the Cleveland dataset available from the UCI repository. They implemented ML algorithms, such as Decision Tree (DT), K-Nearest Neighbor (KNN), NB, and RF for visualizing the probability of heart disease in the patients. Among all models, KNN shows the highest accuracy score of 90% in predicting heart disease.

Garg et al. (15) used an ML algorithm to predict and classify diseases of a person who is suffering from heart problems. They have used a dataset from Kaggle, which was commonly used for prediction purposes. From the attributes, 13 main attributes, such as the age of a person, chest pain, cholesterol level, and many more, were considered to predict heart diseases. The ML algorithms used are KNN and RF. The comparison was made between the ML algorithm and RF achieves the highest accuracy with 82%. Yadav et al. (16) proposed an optimization model, namely, “Optimized DNN using Talos” deploy ML classifiers, such as DT, KNN, RF, and Ensemble model (used ANN, KNN, and Support Vector Machine, SVM), for the prediction of heart diseases. They have used the concept of dimensional reduction where only vital information of patients was considered. On comparing their model with traditional models, the proposed model provides better accuracy and prediction.

Latha and Jeeva (17) convey classification as a commonly used technique in ML algorithms, so they have tried a new technique that is an ensemble technique that combines two or more classifiers to improve the accuracy obtained by a simple classification technique. They focus on increasing the accuracy and implementation of feature selection with a different set of features. There was a significant increment in the prediction accuracy. Singh et al. (18) proposed a multisurface proximal SVM (MPSVM)-based DT, collaborated with the ensembling method consisting of Gradient Boosting and RF algorithms on Cleveland dataset from UCI repository. They have solved the 2-class problem and the 5-class problem on a dataset using the proposed method. On analyzing, the 2-class problem shows better accuracy of 91% than the 5-class problem with 73%. Sowmiya and Sumitra (19) used the Cleveland dataset from the UCI repository to implement heart disease prediction. They have applied an ant colony optimization technique for the best feature selection for the hybrid KNN classifier. This hybrid model is compared to other classification algorithms, such as SVM, KNN, NB, C4.5, and DT. Their study proposed HKNN as an effective and efficient technique for heart disease prediction.

Alotaibi (20) focused on improving the performance and accuracy of ML algorithms compared with the previous studies. In this study, the prediction of heart diseases is made using RapidMiner tool on Cleveland dataset extracted from UCI repository and compared the results with previous work that used Weka and MATLAB tools where the performance of RapidMiner tool gives higher accuracy using techniques: SVM, DT, and Logistic regression.

Nikookar and Naderi (21) proposed a hybrid ensemble method to show comparison with the primary ensemble method using SPECT heart disease dataset, consists of SPECT images based on feature vector on fuser classifier algorithm, i.e., Adaboost, MLP, Logitboost, and RF. The comparison was made between hybrid and basic ensemble classifiers where the proposed hybrid classifier shows higher accuracy. Tama et al. (22) proposed a model using a two-tier ensemble technique to detect heart disease. The developed model was implemented by ensembling gradient boosting, RF, and extreme gradient boosting (XGBoost) classifiers. The model was trained and validated on available datasets: Cleveland, Statlog, Hungarian, and Z-Alizadeh Sani. A two-step significance test was conducted to compare with the existing article and provided the highest results. Li et al. (13) developed a diagnosis system called Fast Conditional Mutual Information (FCMIM-SVM) based on classification algorithms—ANN, SVM, and Logistic Regression—to diagnose heart diseases using the Cleveland dataset. They used minimal redundancy maximal relevance, relief, most minor absolute shrinkage selector operator, etc., as a feature selection algorithm to select relevant features to get accurate accuracy. The proposed model shows a better accuracy as compared to the previously developed model. Sarmah (8) proposed a model using IOT sensors attached to the body of the patient to gather real-time data and applied the Deep learning modified neural network (DLMNN) model for heart disease prediction. The prediction from the model executes in three ways: Authentication, Encryption, and Classification. Thus, the classification shows normal and abnormal output and prescribes it in its manner. Hence, the model proposed shows improvement over the existing algorithm with providing authentication.

Chauhan et al. (10) used an ML algorithm to predict heart diseases on the Cleveland dataset publicly available and compared the result with the algorithms. Among all, ANN outperforms all classification algorithms with 85% accuracy. Pan et al. (14) proposed an enhanced deep-learning Convolution Neural Network (CNN) model that predicts and improves heart disease in a patient. This model uses the more profound architecture of the multilayer perceptron model and regularizes parameters. In addition, this system has been executed on the Internet-of-Medical-Things (IOMT) platform to provide efficient solutions for a doctor for the diagnosis of heart diseases. This CNN model is compared with other ML techniques and shows a maximum accuracy of 97%. Fitriyani et al. (7) developed a clinical decision support system (CDSS) to recognize early heart failure. They also proposed the Heart Disease Prediction Model (HDPM) for the CDSS system to eliminate noise and outlier presence. They have built the Starlog and Cleveland dataset model, which is available publicly and tested on other ML models and compared the results. The proposed model achieves 95% with Statlog and 98% with the Cleveland dataset.

Tate and Rao (23) give an idea of using wearables in form of a wristwatch, wrist band, ambulatory devices, skin response monitors, Fitbit tracker with an app installed, and many more for prediction of cardiac arrest at an early stage. These electronic gadgets help in tracking our day-to-day routine and monitoring our daily activities by keeping track of each activity. The vital information of patients can assist individuals by sending emergency alerts via web and mobile interfaces. Several studies, such as Framingham Heart Study, had developed the Cox regression hazard ratio model specifically for the sex groups for the prediction of heart diseases by estimating 10-year risk. Hence, the idea of capturing the vitals of an individual from wearables helps to get a better prediction and classification rates for detecting heart diseases. Prabhu et al. (24) proposed the CN-Based Multimodal Disease Risk Prediction (CNN-MDRP) model for risk prediction of diseases on a sizeable Medical dataset and compared the result with the algorithm that is CNN-UDRP. The performance of CNN-MDRP shows more improvement than the CNN-UDRP model for risk prediction. Shankar et al. (25) aim to predict heart diseases in an individual. They have made the prediction based on details entered by a patient in a hospital, and the data were trained on a CNN model to find the accuracy. The data used were in structured and unstructured formats. The proposed CNN model shows improvement by comparing it with other algorithms.

Singh and Kumar (12) have calculated accuracy for predicting heart diseases using ML algorithms. The algorithms were KNN, DT, Linear Regression, and SVM utilizing Cleveland dataset from the UCI repository for training and testing. Overall, the accuracy of the KNN algorithm achieves 87% for early heart disease prediction. Amin et al. (26) have collected real-time data, i.e., the vital signs of a patient using Fitbit wearable. The data captured by the wearable were entered into an application and processed through an ML algorithm. This study aims to notify an individual whether he/she is at risk of CVD or not. The outcome of the prediction result helps doctors in diagnosis patient health. Chen et al. (27) proposed a technique for predicting the occurrence of perioperative heart diseases. The data were collected from the hospital and divided into structured data in numerical form and unstructured data in textual form. ML algorithms for numerical data gradient boosting tree algorithm and textual data topic model of text-based data model were used. The datasets were fused using a simple logistic regression model and achieved a sensitivity of 90% and specificity of 93%.

Cohen et al. (28) proposed a 1-year risk prediction model for patients with congenital heart diseases. The dataset was collected from Quebec hospitals of patients varying in age between 18 and 64 years. Multivariate logistic regression was used to predict the risk for heart diseases. The risk score was indicated between 0 and 19. Hence, the risk score model shows excellent performance. Chang et al. (29) use an ML classification algorithm to predict the vital signs of a patient for the next hour. Vital signs, such as heart rate (HR), BP, mean arterial pressure, and oxygen, were considered. Early warning signs can help nurses and doctors treat the patient before the situation degrades. ML algorithms, such as ANN, RF, Gradient Boosting, and long-short-term-memory (LSTM), were developed. The outcome shows the accuracy of ML models. Youssef Ali Amer et al. (30) have carried our experiment with vital signs of patients using wearable devices. The vital signs considered were BP, oxygen level, and HR. These vital signs were estimated every minute using mean and minimum statistical values.

Then, the ML technique of KNN LS SVM was executed to predict future values of vital signs that were monitored. The performance of implemented ML algorithm was compared with the LSTM approach, and the proposed approach shows much improvement in predicting the early warning score of vital signs. Stehlik et al. (31) have indicated re-hospitalization of HF by non-invasive and remote monitoring. Wearable sensors were placed on the chest of a person (up to 3 months), collecting and recording physiological data (vital signs). The data were uploaded on a cloud platform via a smartphone. Analytics were performed on data to detect HF exacerbation. The result shows 76–88% sensitivity and 85% specificity. An alert was shown 6 days prior for re-admission.

Jia et al. (32) use the Cox proportional hazards regression model to find the probability of risk for 10 years using the Framingham dataset. The risk factors considered were sex, body mass index (BMI), systolic BP (SBP), and diabetes. They have calculated a sex-based risk score using the Cox regression hazard equation.

Yang et al. (33) proposed a multivariate regression model for the prediction of CVDs. The prediction was made on the collection of vital information of patients from the centers of the national high-risk program by having a cardiac events assessment. The dataset consists of all information, such as BP, cholesterol level, obesity, smoking, BMI, and many more attributes were taken into consideration. The area under curve results of the multivariate regression model were then compared to other ML classification techniques, such as CART, NB, and Ada Boost. The outcome result of the multivariate regression model outperforms other methods. Made et al. (34) surveyed CVDs estimating 10-year risk in the different age groups of people. They proposed a SCORE model that uses risk factors, such as weight, BP, cholesterol level, smoking habit, diabetes, and many more. The data were collected by taking samples of men and women of age 18 and above. The 10-year risk estimation was made on statistical analysis, which estimates coronary heart diseases and no-coronary heart disease. These studies on 10-year risk scores help to prevent heart diseases in the coming future.

### Comparison of Previous Findings and Proposed Study

The literature study discussed above and in Table 1 shows the importance of the vital signs of a person in the healthcare industry to detect health-related problems. Mainly, health problems are majorly concerned with the heart, such as cardiac arrest, CVDs, coronary heart disease, and many more. In this research, the authors have depicted the detection of cardiac arrest in a person by collecting real-time data. Moreover, most of the detection and prediction of heart disease is majorly made on a public dataset consisting of shared attributes. In our contribution, the features chosen are mainly focused on cardiac arrest detection. After detecting cardiac arrest, gender-wise survival probability, and age-wise cardiac arrest risk scores are also formulated using two approaches: (1) Kaplan-Meier and (2) Cox regression model. These two methods help to provide which gender has fewer survival chances and which age group has a high probability of risk in the next 10 years.

**Table 1:** Comparison of literature work and proposed work.

**References**	**Findings**	**Method used**	**Results obtained**
Ramprakash et al. (2)	Prediction of heart disease on Cleveland dataset	Machine learning algorithms—Deep Neural Network and χ^2^ —a statistical method	Accuracy—94%
Mohan et al. (6)	Prediction of heart disease on Cleveland dataset	Proposed HRFLM model using Random Forest, and Linear model	Proposed model accuracy—88%
Sowmiya and Sumitra (19)	Used Cleveland dataset to implement heart disease prediction	Used ant colony optimization technique for hybrid KNN classifier and compared with other ML models	Proposed approach HKNN proves efficient and effective technique
Made et al. (34)	Estimation of 10-year coronary heart risk	Using statistical approach—regression analysis in age group of 18 and above	Study estimates heart risk for the next 10-years
Proposed work	Detection and classification of cardiovascular diseases in different age-based group and gender-based people, collected vital signs of an individual using wearables and medical equipments	Used 6 Machine Learning algorithm—Decision Tree, SVM, ANN, RFC, XGBoost, and NB. Further, comparison is made among 6 classifiers. Also, we developed two models based on Kaplan-Meier method (For Gender-Based) and Cox-Regression Proportional Model (For Age-Based) using scoresheet	Among 6 Machine learning models- Decision Tree achieves maximum accuracy of 98%. Our proposed approach, for age-based and gender-based shows risk score (in percentage) of cardiac arrest for the next 10-years

## Proposed Work

This section briefly describes the novelty of the proposed work (contributions) and the articles mentioned early by some research on the same Cox regression model.

The progress in CVD risk models is ordinarily based on conventional laboratory-based predictors. The primary purpose was to develop a prediction model, which was based on a 10-year risk. To predict 10-year risk, a Cox regression proportional method was applied. A dataset from Framingham Original Cohort and SCORE of men and women aged between 30 and 62 was taken to predicate CVD risk. In this study, the CVD risk was being predicted on a gender basis (men/woman). The risk factors involved are age, sex, BMI, SBP, and diabetes.

A proposed study related to cardiac arrest prediction using the real-time dataset is classified based on gender and age. Our study has used two methods to find the risk classification probabilities among gender-based and age-based. The two methods used—Kaplan-Meier survival analysis and Cox regression model survival analysis—are considered. These two methods have their importance in analyzing risk probabilities.

### Kaplan-Meier Method

This method is a non-parametric survival function for the estimation of survival probability. Kaplan-Meier method also estimates the survival curve by giving a statistical comparison between two groups: men and women.

### Cox Regression Proportional Model

Cox regression proportional model does regression analysis with survival data without making any strong assumption. It calculates the hazard ratio (HR) of the covariates used in our data and is based on the equation, and it shows risk probability. Cox regression proportional model is a model that helps to find risk probability or score on more than one covariate. In general, it is a method to identify the effect of variables at some time interval on some event that occurs.

## Methodology

### Measuring Device

The risk factors associated with heart diseases are cardiac arrest prediction: age, gender, height, weight, BMI, BP [SBP and diastolic BP (DBP)], HR (beats per minute), and oxygen level (SPO_2_%).

**Figure 1:**
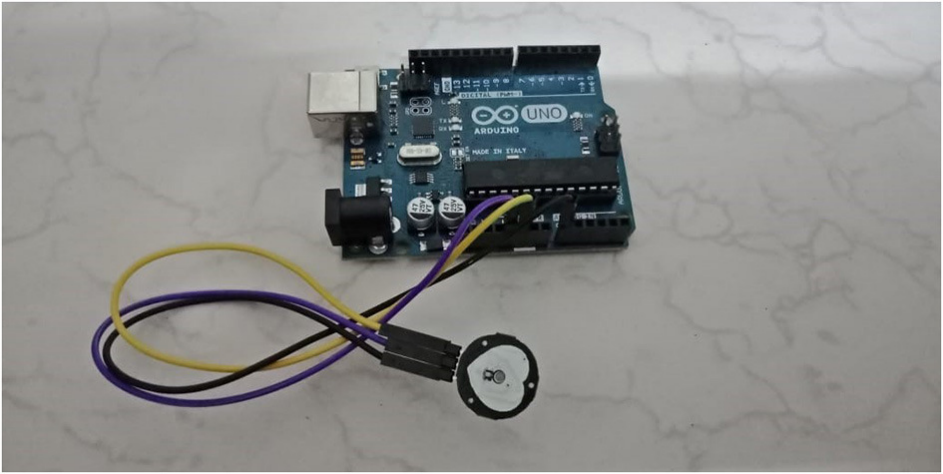
Pulse sensor connected with Arduino.

**Figure 2:**
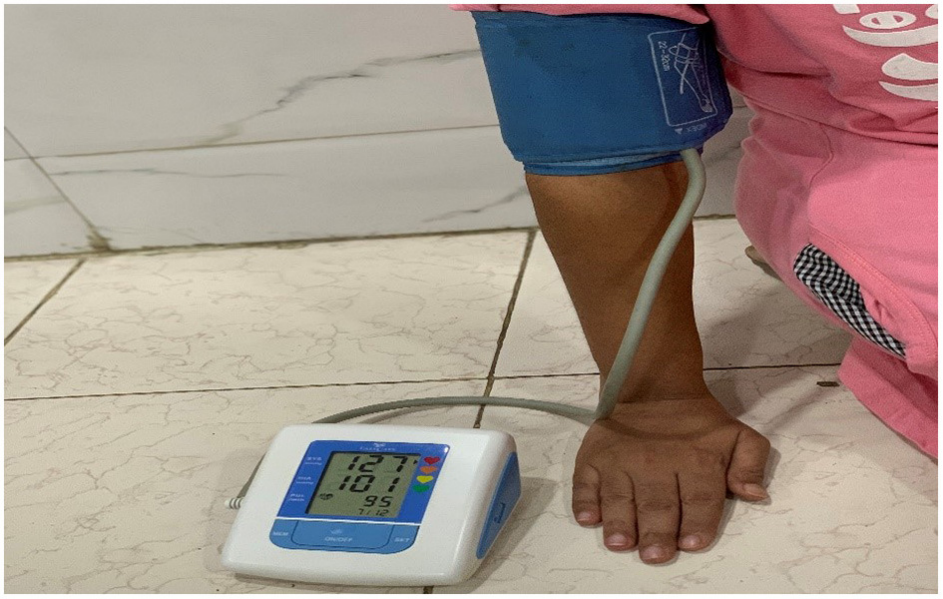
Sphygmomanometer.

**Figure 3:**
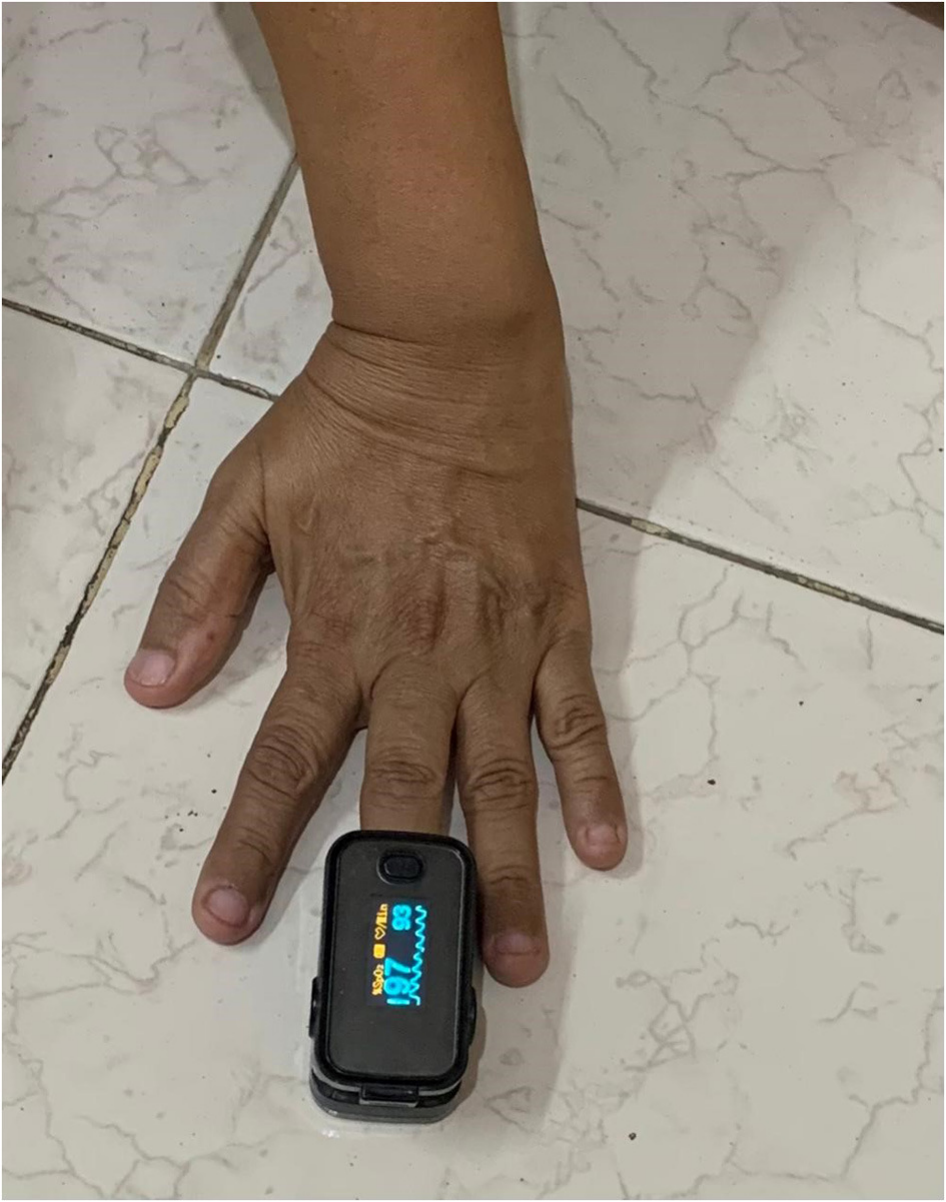
Oximeter.

The above factors used in this research are collected using IOT sensors and equipment for different risk factors. The measuring device can capture the vital signs of a person needed to estimate cardiac arrest prediction. Figures 1–**3** show measuring device used for capturing vital signs.

#### Data Collection

##### Pulse Sensor

A pulse sensor is a device that is used to detect biometric pulse rate or HR, and it also keeps track of volume change. It is generally helpful for healthcare applications to capture the pulse rate of an individual. A pulse rate is detected when there is a change in blood vessel volume, and that occurs when the heart pumps blood.

For this research, we used a pulse sensor that is connected to Arduino UNO Board. Above, Figure 1 shows the configuration of the pulse sensor and Arduino Board. A pulse rate or HR was captured from a total of 18 patients in beats per minute (BPM) at a 2 min interval.

##### Sphygmomanometer

A sphygmomanometer is a device used to measure the BP of a person. The connected rubber cuff with the device is wrapped around the upper arm of a patient and records the blood pressure in SBP and DBP.

For this research, we used Sphygmomanometer to measure BP. Above, Figure 2 shows a Sphygmomanometer connected to the arm of a patient. BP was captured from the same 18 patients at every 2 min interval.

##### Pulse Oximeter

The pulse oximeter is a measuring device used to keep track of oxygen levels in a human body. This is an easy and painless measuring device to see how much oxygen reaches the different parts of the body. This device is generally used in hospitals and at home to determine the oxygen level in the body. It measures the oxygen level in SPO_2_% form.

For this research, we used a pulse oximeter to measure the SPO_2_. Above, Figure 3 shows a pulse oximeter placed on the fingertip of a patient and the estimated oxygen level. It is a non-invasive method for monitoring oxygen saturation. Oxygen level was captured from the same 18 patients at every 2 min interval.

#### Dataset Description

Table 2 shows the names of attributes along with their feature description. The table also contains a range column indicating the range value mentioned in our dataset and its dataset. The dataset comprised attributes are:

- Date and time
- Gender
- Age
- Height
- Weight
- BMI
- SBP
- DBP
- HR (BPM)
- Cardiac arrest prediction—target variable.

**Table 2:** Dataset description.

**Sr. No**.	**Attributes name**	**Description**	**Value range**	**Type**
1	Date and time	Shows person data collected on date and time	–	DD-MM-YYYY hh:mm:ss
2	Gender	Shows gender of a person	Male = 1 Female = 0	Categorical
3	Age	Shows age of a person in years	Age from 15 to 75	Numerical
4	Height	Shows height of a person in feet and inches	Height varies from 4′3′′-5′9′′	Numerical (in feet and inches)
5	Weight	Shows weight of a person in kilogram (kg)	Weight ranges from 45 to 75 kg	Numerical (in kg)
6	Body Mass Index (BMI)	Shows body mass of a person	BMI ranges from 15.1 to 38.7	Float
7	Systolic blood pressure (BP)	Shows systolic blood pressure of a person in mmHg	Normal Range must be ≥120 to ≤ 134 mmHg	Numeric
8	Diastolic blood pressure (BP)	Shows diastolic blood pressure of a person in mmHg	Normal range must be ≥79 to ≤ 87	Numeric
9	Heart rate (BPM)	Shows heart rate of a person in beats per minute	Heart rate between ≥49 to ≤ 84	Numeric
10	Oxygen level (SPO_2_)	Shows oxygen level in a person body	Range between ≥95% to ≤ 100%	Numeric in %
11	Cardiac arrest prediction	Target variable to be predicted	0 = No cardiac arrest 1 = Cardiac arrest	Binary value

Cardiac arrest prediction is our target variable from the features mentioned above, which is predicted in our dataset.

The target variable—cardiac arrest prediction—is labeled using the following conditions, as mentioned in Table 3.

**Table 3:** Labeled target variable.

**Parameter**	**“0”—predicted label**	**“1”—predicted label**
Systolic blood pressure mmHg	Between ≥120 to ≤ 134 mmHg	Below 120 mmHg and above 134 mmHg
Diastolic blood pressure mmHg	Between ≥79 to ≤ 87 mmHg	Below 79 mmHg and above 87 mmHg
Heart rate (BPM)	Between ≥49 to ≤ 84	Below 49 and above 84
Oxygen level (SPO_2_)	Between 95–100%	Below 95% or above 100%

### Data Pre-processing

In the ML process, the processing of datasets is essential for good impersonation. Some techniques for data pre-processing are data cleaning, data integration, data transformation, data reduction, and data discretization. In our dataset, there are 540 records where the column gender is denoted as “F” and “M.” These need to be processed as “0” and “1” using LabelEncoder.

### Algorithm Used

#### Artificial Neural Network

Artificial Neural Network consists of ample neurons simulated together to form a network architecture. This architecture of the neural network is used to transform the inputs into some meaningful outputs. ANN is a supervised ML technique generally used for prediction. ANN consists of an input layer, hidden layer, and output layer linked together, forming a network. These layers are associated with numerical weights to minimize the error mathematically. For the cardiac arrest prediction dataset, the input layer consists of SBP, DBP, HR, and oxygen levels, fed to 16 neurons. These input parameters are fed into the next layer. The hidden layer consists of eight neurons and finally, after the processes, gives output to the output layer as a final result.

#### Random Forest Classifier (RFC)

Random Forest Classifier is a type of supervised ML algorithm used for two purposes: classification and regression problems. It follows the concept of ensemble learning, which combines multiple classifiers to solve complex problems and improves the performance of a model. Generally, it consists of several DTs forming a subset of a given dataset and improving the predictive accuracy of a dataset. RFC predicts the final output by comparing the prediction from each tree and based on the decision, such as majority voting; it indicates the result. For the cardiac arrest prediction dataset, the input parameters considered are SBP, DBP, HR, and oxygen level. The dataset is divided into 80% training and 20% testing set and selected the number of trees estimator 30 and function “entropy” for the information gain.

#### Gradient Boosting (XGBoost)

“XGBoost” stands for extreme gradient boosting and can be implemented with the gradient boosted trees algorithm. XGBoost is a supervised ML algorithm and is considered one of the most sought-after ML techniques. This is due to its high prediction accuracy and user-friendliness. Just like other ML algorithms, XGBoost can also be used for regression and classification of problems. XGBoost uses DTs as a weak predictor and minimizes the loss function using a gradient descent algorithm. In implementation, we created an “XGB Classifier” object by passing parameters subsample = 0.7, max_depth = 5, and n_estimators = 100 and by default it takes booster = “gbtree.” The dataset is divided into 80% training and 20% testing set where training data are used to train a model and test part to measure its performance.

#### Support Vector Machine

Support Vector Machine is the best-supervised ML algorithm. It is used for both regressions and classification problems. However, for the dataset used, this algorithm is used for classification problems. For the cardiac arrest prediction dataset, the input parameters considered are SBP, DBP, HR, and oxygen level. The data are divided into 80% training and 20% testing set. We have used the Kernal value “rbf” Radial Basis Function for a non-linear classification problem.

#### Naïve Bayes

Naïve Bayes is a supervised ML algorithm that is used for solving a classification problem. It is generally based on the Bayes theorem and is considered one of the best classification techniques. It makes a quick prediction based on object probability. It builds a model on the classification problem and shows the high accurate result for a large dataset. For the cardiac arrest prediction dataset, the input parameters considered are SBP, DBP, HR, and oxygen level. The data are split into 80% training and 20% testing set. The model used in making a prediction is the Gaussian NB model, which follows a normal distribution for our predictor values.

#### Decision Tree

A DT is a classification technique used to solve a classification problem. It is a simple and widely used classification method. It is generated by providing a set of inputs in the form of a dataset. Its structure is like a flowchart that consists of Root, Sub-tree, and Leaf nodes. The algorithm starts from the root node, compares the values with the dataset attribute, and on the comparison, it follows the tree structure, i.e., branch and jumps to the next node. It continues its process until it reaches the last node, i.e., a leaf node. This helps in predicting the class of the given dataset. For the cardiac arrest prediction dataset, the input parameters considered are SBP, DBP, HR, and oxygen level. The data are split into 80% training and 20% testing set, considering the maximum depth of tree-level to be three and attribute used “Gini” to measure purity while creating a DT.

## Risk Classification

Survival analysis is a statistical approach for analyzing data where the outcome variable is the time till an event occurs. They are categorized into parametric, semi-parametric, and non-parametric models. For analyzing data, several survival analysis techniques are used, such as Life Tables, Kaplan-Meier analysis, Cox proportional hazards regression model, Survival trees, and Survival random forest. In this section, the different methods we used for our survival analysis are introduced.

### Risk Survival Analysis Model

In this study, two models are proposed: Age_Cox_model specific for age-based survival analysis and Gender_KM_model for gender-based survival analysis. Both models are used to find probability risk in an individual. Age_Cox_model is generally based on the Cox regression model to predict the survival probability of having different ages. Gender_KM_model is based on the Kaplan-Meier estimator to analyze the survival probability based on male and female groups.

#### Age-Wise Classification

The age-based classification is done using Age_Cox_model analysis. The first stage of Age_Cox_model is to provide input in the form of a dataset. The next step is the pre-process the dataset to solve the categorical data such as gender. For categorical problems, the use of LabelEncoder is applied for the gender feature. The third stage is to select relevant parts for our proposed model. After selecting features, the next step is to train our model by applying the Cox regression model. The model then predicts the result, showing the importance of features and whether there are any risks. The steps of our model are described in the following pseudocode.

Step 1: Import libraries,
Step 2: Load the dataset,
Step 3: Pre-processed the data,
Step 4: Select relevant features,
Step 5: Apply Cox regression CoxFit() model,
Step 6: Predict results show feature importance and chances of risk.

**Figure 4:**
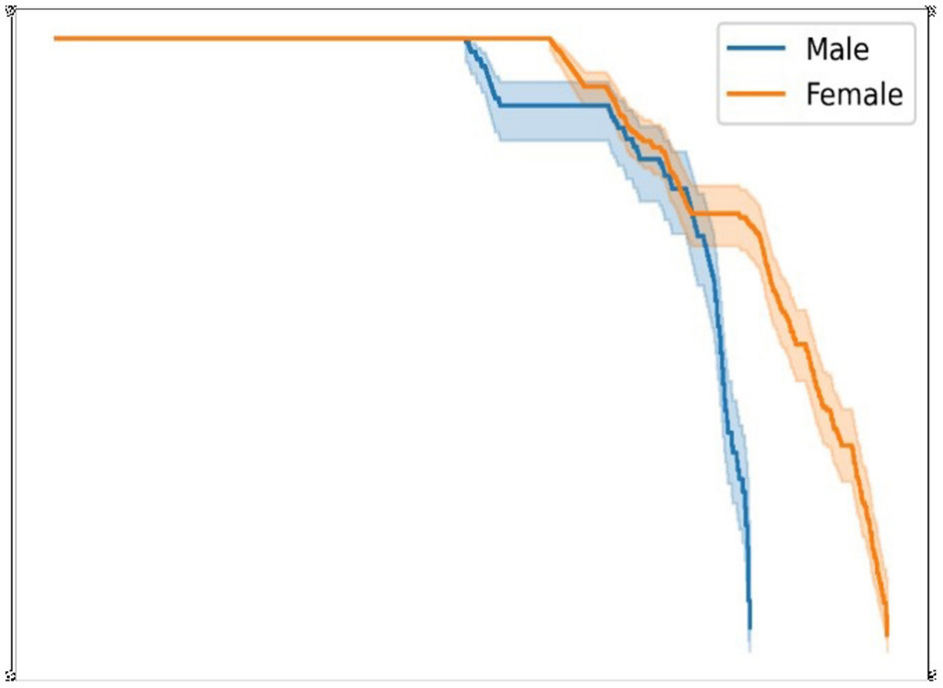
Age_Cox model.

The flow of the work is presented in the below diagram, Figure 4.

The above diagram result shows the importance and risk probability of features. Table 4 describes that the *p*-value must be <0.05 and is considered significant.

**Table 4:** Summary of Age_Cox_model.

	***β***	**Exp(*β*)**	**Se(*β*)**	***β* lower 95%**	***β* upper 95%**	**Exp(*β*)** **lower 95%**	**Exp(*β*)** **upper 95%**	***z***	***p***	**–log_2_(*p*)**
Age	0.03	1.03	0.00	0.02	0.03	1.02	1.03	7.32	<0.005	41.86
Systolic BP	−0.04	0.96	0.01	−0.05	−0.03	0.95	0.97	−7.35	<0.005	42.25
Diastolic BP	0.01	1.01	0.01	−0.01	0.02	0.99	1.02	0.72	0.47	1.09
Heart rate (BPM)	0.00	1.00	0.00	0.00	0.01	1.00	1.01	2.88	<0.005	7.98

The *p*-value of attributes age, SBP, and HR (BPM) is considered a significant covariant as values are <0.05.

On considering HR, i.e., exp(coef) shows the level of risk and have the following condition:

If HR = 1 Covariant has no effect of risk
HR <1 Covariant has fewer chances of risk
HR >1 Covariant has more options of risk.

Hence, to know the chances of risk in our proposed work, age-based classification can be solved using the Cox regression survival analysis model formula, which is explained in the next part.

**A.** **Age-based risk score calculation**

The age-wise classification is derived from the prediction of a 10-year risk score for cardiac arrest prediction. Among the features collected, the main risk factors for cardiac arrest prediction are a Person's age, BP (SBP and DBP), HR (measured in BPM), cardiac arrest prediction as target variable, and T1 factor as a time interval. The age-wise classification for 10-year risk probability is estimated using the Cox regression model.

Table 5 represents the characteristics of risk factors that show statistical values of “total number of observations” (in each column), “number of observations with missing values,” “number of observations without missing values,” “minimum value” (in each variable column), “maximum value” (in each variable column), “mean value” (in each variable column), and “SD value” (in each variable column) are encapsulated.

**Table 5:** Summary of statistical values of risk factors used for 10-year risk model.

**Feature**	**Obs**.	**Min**	**Max**	**Mean**	**Std.** **deviation**
T1	540	672.00	1363.00	1076.831	167.757
Cardiac arrest prediction	540	0.00	1.00	0.607	0.489
Age	540	15.00	75.00	43.056	19.364
Systolic BP	540	82.00	157.00	118.961	14.896
Diastolic BP	540	55.00	125.00	78.113	9.905
Heart rate (BPM)	540	24.00	238.00	87.006	39.929

Table 6 shows the summary statistics of events that occur while modeling risk prediction for 10 years. It shows, “total observed” = 540 total number of data or observation, “total failed” = 328 denotes the number of possible cardiac arrests, “total censored” = 212 denotes thin chances of cardiac arrest, and “Timestep” = 540 denotes the number of time steps equals to the total number of observations.

**Table 6:** Summary statistics (events).

**Total** **observed**	**Total** **failed**	**Total** **censored**	**Time** **steps**
540	328	212	540

The following table indicator, Table 7, represents the model quality, that is, “efficiency of the model/variable fit.” The most important statistic value to be noted is the probability of the Chi-square test on the log-ratio as shown in Table 8; on comparing, the model statistics with the defined covariates, the probability value, that is, the *p*-value must be lower than 0.05 (*p* < 0.05), to be significant. Hence, from Table 7, we conclude that the variables show significant information.

**Table 7:** The goodness of fit statistic.

**Statistic**	**Independent**	**Full**
Observations	328.000	328.000
DF	0.000	4.000
−2 Log (likelihood)	3487.800	3398.055
AIC	3487.800	3406.055
SBC	3487.800	3421.227
Iterations	1.000	3.000

**Table 8:** Test of the null hypothesis H0: beta = 0.

**Statistic**	**DF**	**Chi-square**	**Pr > Chi^2^**
−2 Log (likelihood)	4	89.74558	<0.0001
Score	4	94.97911	<0.0001
Wald	4	94.87288	<0.0001

The following table gives information on the model. Table 9 helps us understand the effect of our risk factors on the model. From Table 9, as per our collected dataset, the variable that influences our risk survival model is SBP. From the beginning of the study, this risk variable indicates the patient has a high effect on cardiac arrest prediction. In this study, from the table, the “Value” column shows the HR, which is obtained as the estimated parameter exponent and is calculated using Equation (1).

**Table 9:** Regression coefficients.

**Variable**	**Value**	**Std. error**	**Wald Chi-square**	**Pr > Chi^2^**	**HR**	**HR <95%**	**HR > 95%**
Age	0.027	0.004	53.587	<0.0001	1.027	1.020	1.035
Systolic BP	−0.039	0.005	54.068	<0.0001	0.962	0.000	0.972
Diastolic BP	0.005	0.007	0.517	0.472	1.005	0.991	1.019
Heart rate (BPM)	0.004	0.001	8.232	0.004	1.004	1.001	1.007

The HR is given using the mentioned formula:


(1) $$\begin{array}{c} \text{H}(\text{t})={\text{h}}_{0}(\text{t})\text{exp}(\sum^{\text{​}}{ß}_{\text{i}}{\text{X}}_{\text{i}})(\text{i}=1\;\text{to}\;\text{r})\;(1) \end{array}$$

Notations are:

t = Survival time (10-year risk) (t = 10).
exp = Exponential function (exp(X)).
B_i_ = It measures the impact of covariates (B_1_, B_2_, B_3_, …, B_r_).
X_i_ = Predictor variables (X_1_, X_2_, X_3_, …, X_r_).
h_0_(t) = Baseline hazard rate.
H(t) = HR.

The Cox model regression has an exponential form, as seen in Equation (1).

As per our dataset, for 10 years, the term h_0_(t) = baseline survival rate value is considered 0.993 at mean values of covariates from the survival distribution function. Here, “t” represents an event that occurred at a time. The H(t) hazard function is determined by the set of covariates X_i_ = (X_1_, X_2_, X_3_, …, X_r_) and the regression coefficient B_i=_ (B_1_, B_2_, B_3_, …, B_r_) that measures the impact of covariates.

In Table 10, the column “rho,” correlates between residuals and time vector as in our above case—Kaplan Meier. The other two columns, “Chi-square” and “Pr > Chi-square,” show test statistics and the associated *p*-value. Here, the *p*-values must be <0.005, and the value from Table 10 reveals no violation of the risk assumption.

**Table 10:** Proportionality test.

**Variable**	**Rho**	**Chi-square**	**Pr > Chi^2^**
Age	−0.22834	24.24338	<0.0001
Systolic BP	0.09274	4.133863	0.042
Diastolic BP	0.012658	0.061134	0.805
Heart rate (BPM)	0.136702	7.842881	0.005
Global		32.0064	<0.0001

Hence, to find the survival risk score from the Cox model regression, it can be written as:


(2) $$\begin{array}{c} \text{R}(\text{t})=1-\text{R}0(\text{t})\;\text{exp}\;(\sum^{\text{​}}\text{ßiXi}-{\sum^{\text{​}}}^{\text{​}}\text{ßi}\bar{\text{X}}\text{i})\;(\text{i}\;=1\;\text{to}\;\text{r}) \end{array}$$

R(t) is the risk estimation of cardiac arrest of an individual; R_0_(t) is the baseline survival time at t; β*i* is the regression coefficient covariates values mentioned in Table 8; X_i_ is the value of the i_th_: the risk factor value [log-transformed value – natural log (ln)]; and ${\bar{\text{X}}}_{I}$is the mean value of the covariates. Here, the i value ranges from 1 to r and denotes the risk factors considered. The cardiac arrest risk score or percent can be determined using the mentioned formula as shown in Equation (2).

The estimation of the Cox regression model is formulated from the scoresheet, and the Cox regression equation is derived. Tables 11, 12 show the scoresheet that represents the points allocated to an individual risk factor for both genders. Table 13 shows the total risk score probability after formulating Equation (2).

**Table 11:** Cardiac arrest risk score for men.

**Male**			
**Points**	**Age**	**Blood pressure**	**Heart rate**
−1	30–34		<50
0	35–39	<130/85	50–70
1	40–44	130/85–139/89	70–90
2	45–49	140/90–159/99	90+
3	50–54	≥160/100	
4	55–59		
5	60–64		
6	65–69		
7	70–74		

**Table 12:** Cardiac arrest risk score for women.

**Female**			
**Points**	**Age**	**Blood pressure**	**Heart rate**
−9	30–34		<50
−4	35–39	<120/89	50–70
0	40–44	120/80–139/89	70–90
3	45–49	140/90–159/99	90+
6	50–54	≥160/100	
7	55–59		
8	60–74		

**Table 13:** Ten-year risk for the Risk points calculated.

**10-year risk score (%)**			
**Points**	**Male**	**Points**	**Female**
−3	1%	< -2	1%
−2 or −1	2%	−1, 0, or 1	2%
0	3%	2 or 3	3%
1 or 2	4%	4	4%
3	6%	5	5%
4	7%	6%	6%
5	9%	7	7%
6	11%	8	8%
7	14%	9	9%
8	18%	10	11%
9	22%	11	13%
10	27%	12	15%
11	33%	13	17%
12	40%	14	20%
13	47%	15	24%
14 or more	56%	16	27%
		17 or more	32%

The calculation for estimation of cardiac arrest risk is derived below:

**B.** **Risk estimation from the scoresheet**

As mentioned in Tables 11, 12, the risk points are calculated according to the covariate's ranges. Table 14 shows points allocated according to the risk factor involved.

**Table 14:** Risk point calculation.

**Risk factor involved**	**Values**	**Points allocated**
Gender	Female	–
Age	50	6
Systolic blood pressure	131	0
Diastolic blood pressure	80	0
Heart rate	112	3
Total points scored		9
Risk estimation according to sheet		9%

**C.** **Risk estimation from Cox regression model**

As per Equation (2), risk estimation of cardiac arrest in female is given by:

Formula:

R(t) = 1 – R0(t) exp (∑ ßiXi – ∑ßi$\bar{\text{X}}$ i) (i = 1 to r)

Values for:

- R_0_(t) for t = 10 years = 0.993 is the baseline survival
- β*i* is the estimated regression coefficient (in Table 9)
- *Xi* is the log-transformed value of ith risk factor
- $\bar{X}$*I* is the mean value of the covariates considered (in Table 5)
- R is the number of risk factors involved in predicting risk. The risk estimation of the cardiac arrest based on the Cox regression model is computed as:
- *Xi* is the log-transformed value of ith risk factor
- $\bar{X}$*I* is the mean value of the covariates considered (in Table 5)
- R is the number of risk factors involved in predicting risk.

The risk estimation of the cardiac arrest based on the Cox regression model is computed as:


$$\begin{array}{l} \sum {\;}_{i=1}^{r}\beta iXi=[0.027^{*}\ln(50)]+[-0.039^{*}\ln\;(131)]\\ \;+[0.005^{*}\ln(80)]+[0.004^{*}ln\;(112)]\\ \;=0.1056--0.1901+0.0219+\;0.0188\\ \;=-0.0438\\ \sum {\;}_{i=1}^{r}\beta i\bar{X}i=[0.027^{*}43.056]+[-0.039^{*}118.961]\\ \;+[0.005^{*}78.113]+[0.004^{*}\;87.006]\\ \;=1.1625-4.6394+0.3905+\;0.3480\\ \;=-2.7384 \end{array}$$

Substituting values in Equation (2) we get,


$$\begin{array}{l} R(t)=1-R_{0}(t)\exp(\sum {\;}_{i=1}^{r}\beta iXi-\;\sum {\;}_{i=\;1}^{r}\beta i\bar{X}i)\\ \;=1-R_{0}(10)\exp(-0.0438-\;(-2.7384))\\ \;=1-(0.993)\exp\;(2.6946)\\ \;=1-(0.993){\;}^{\left(14.79\right)}\\ \;=1-\;0.9013\\ \;=0.0987\\ \;=0.0987^{*}100(\text{convertingto}\;\%)\\ \;=9.87\\ \;\approx 9\% \end{array}$$

According to the scoresheet, the point system is 9%, and the Cox regression model also gives the approximate percentage of risk 9%. Therefore, a female having age 50 may have a chance of 9% more risk of cardiac arrest in the coming 10-year risk prediction.

#### Gender-Wise Classification

The gender-based classification is done using Gender_KM_model analysis. The first stage of Gender_KM_model is to provide input in the form of a dataset. The next step is to pre-process the dataset to solve the categorical data such as gender. For categorical problems, the use of LabelEncoder is applied for the gender feature. We divide our dataset into two groups: male and female and apply the Kaplan-Meier analysis model. The model then predicts the result, showing prediction probability in each gender at an event time. The steps of our model are described in the following pseudocode.

Step 1: Import libraries
Step 2: Load the dataset
Step 3: Pre-processed the data
Step 4: Divide dataset into two groups: male and female
Step 5: Apply Kaplan-Meier KaplanMeierFitter() model
Step 6: Predict results shows probability in each gender at an event time.

**Figure 5:**
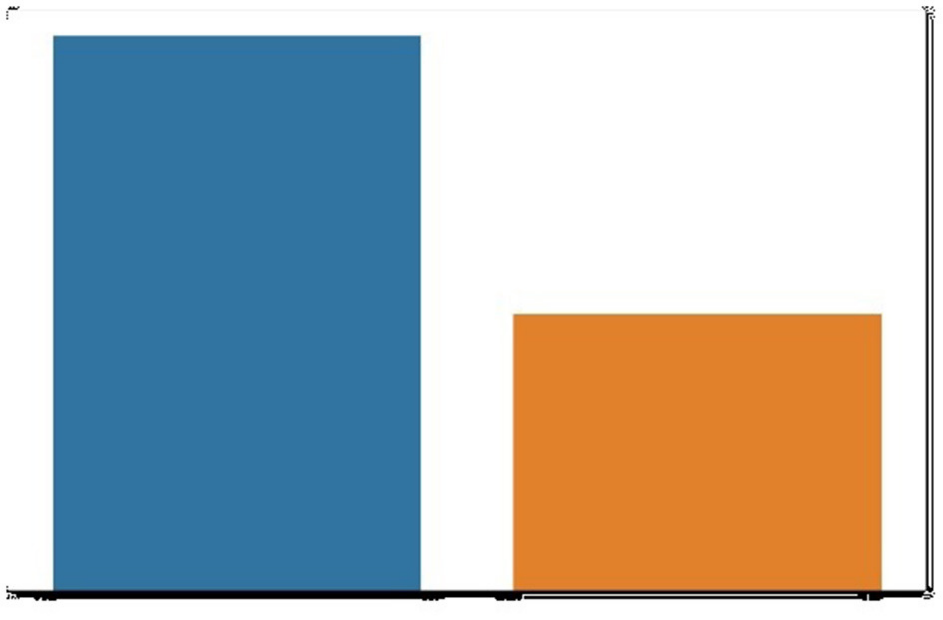
Gender_KM model.

The flow of the work is presented in the below diagram, Figure 5.

**A.** **Gender-based estimation of risk probability**

The gender-wise risk classification can be estimated using a non-parametric survival estimator called the Kaplan-Meier method. It is useful in determining the survival probabilities of an individual and gender-wise. As per our collected dataset, survival function estimator S(t) can be given as:


(3) $$\begin{array}{r} \text{S}\left(\text{t}\right)=\text{Π}\left(1-\text{di}/\text{ni}\right)\left(\text{i}:\text{ti}<=\text{t}\right) \end{array}$$

Where,

S(t) = gives survival probability
t_i_ = time at when an event occurs
d_i_ = number of events occur (e.g., at risk of cardiac arrest) at time ti
n_i_ = number of individuals who have survived (e.g., not at risk of cardiac arrest) at time t_i_

The Kaplan-Meier estimator helps to find the probability or survival rates of adjusted covariates. In our dataset, our estimation is to classify risk according to gender (male and female). We have divided our data into two groups: male and female. Our target is to notice any significant difference in probability or survival rate if data are divided into two groups based on gender. Based on gender split, we come up with survival probability in men and women based on Equation (3), which is shown in Tables 15, 16.

**Table 15:** Event table for male.

**Event_at**	**Removed**	**Observed (d_1_)**	**Censored**	**Entrance**	**At_risk (n_i_)**
0	0	0	0	180	180
672	1	1	0	0	180
674	1	0	1	0	179
676	1	1	0	0	178
678	1	1	0	0	177
680	1	1	0	0	176
682	1	0	1	0	175
684	1	0	1	0	174
686	1	0	1	0	173
688	1	1	0	0	172
690	1	1	0	0	171
692	1	0	1	0	170
694	1	0	1	0	169
696	1	1	0	0	168
698	1	1	0	0	167
......	......	......	......	......	......
1,120	2	1	1	0	20
1,122	2	0	2	0	18
1,124	2	0	2	0	16
1,126	2	1	1	0	14
1,128	2	0	2	0	12
1,130	2	1	1	0	10
1,132	2	1	1	0	8
1,134	2	1	1	0	6
1,136	2	2	0	0	4
1,138	2	1	1	0	2

**Table 16:** Event table for female.

**Event_at**	**Removed**	**Observed (d_i_)**	**Censored**	**Entrance**	**At_risk (n_i_)**
0	0	0	0	360	360
810	1	1	0	0	360
812	1	1	0	0	359
814	1	1	0	0	358
816	1	1	0	0	357
818	1	1	0	0	356
820	1	1	0	0	355
822	1	1	0	0	354
824	1	0	1	0	353
826	1	1	0	0	352
828	1	1	0	0	351
830	1	1	0	0	350
832	1	1	0	0	349
......	......	......	......	......	......
1,345	2	1	1	0	20
1,347	2	2	0	0	18
1,349	2	1	1	0	16
1,351	2	1	1	0	14
1,353	2	1	1	0	12
1,355	2	1	1	0	10
1,357	2	1	1	0	8
1,359	2	0	2	0	6
1,361	2	1	1	0	4
1,363	2	1	1	0	2

The survival probability/risk of an individual (male/female) is estimated based on the time interval. On predicting survival probabilities in both genders using object creation of Kaplan-Meier we get,

kmf_m.predict (900) = 0.89061… ≈ 89%
kmf_f.predict (900) = 0.92205… ≈ 92%

Here, predict (900) indicates predicting survival or risk probability at 900-time intervals. The male probability of surviving is 89%, and the female probability of surviving is 92%.

**Figure 6:**
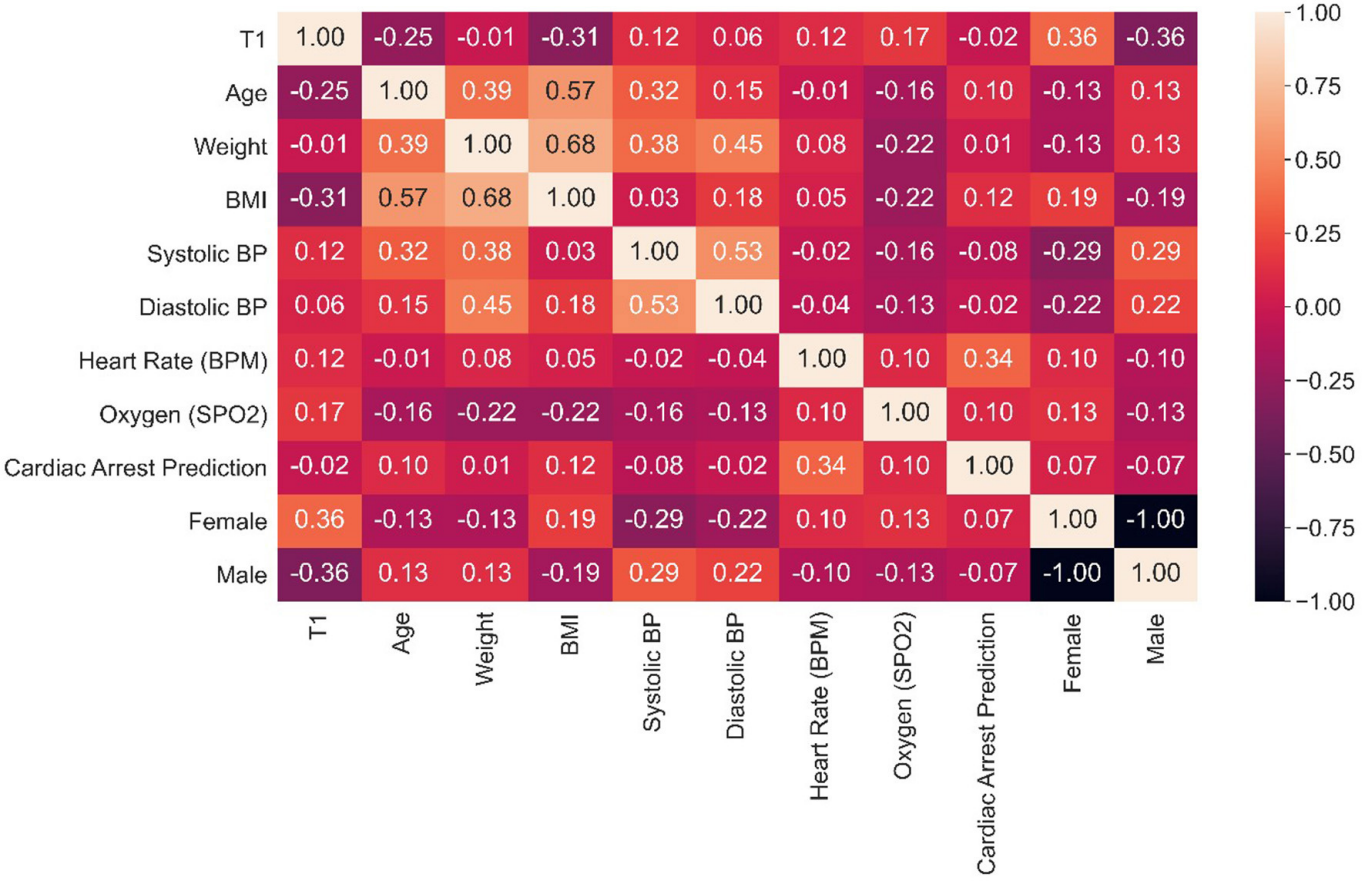
Men/women survival probability graph.

From the above observation, we can conclude that the survival probability of women is higher compared to men. Men are at higher risk than women. Below, Figure 6 shows the survival probability of men and women at a given time t.

## Exploratory Data Analysis

**Figure 7:**
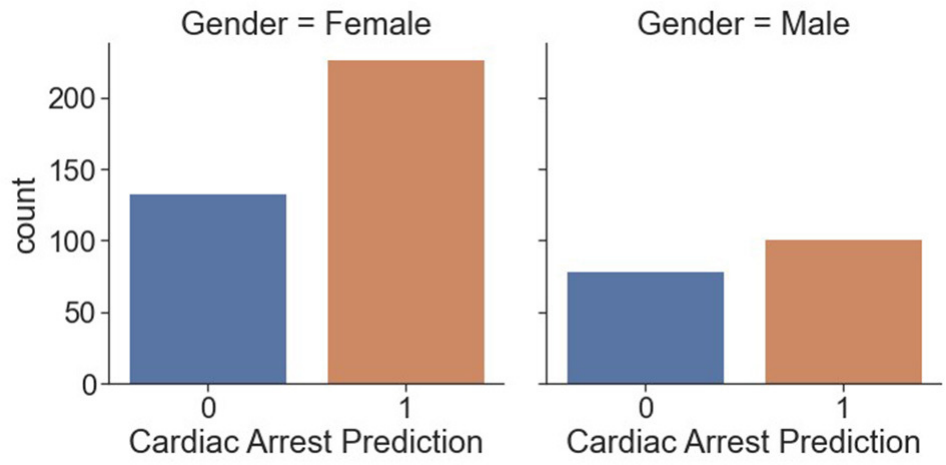
Counts of men and women.

Figure 7 shows the number of female and male data in our dataset from the figure. We can conclude that there are 150 “men” and 350 “women.”

**Figure 8:**
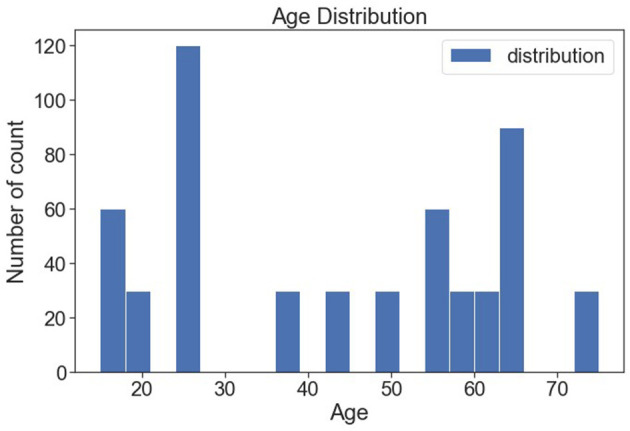
Attributes correlation matrix.

Figure 8 shows a correlation graph among attributes, using the syntax:

data.corr( )

**Figure 9:**
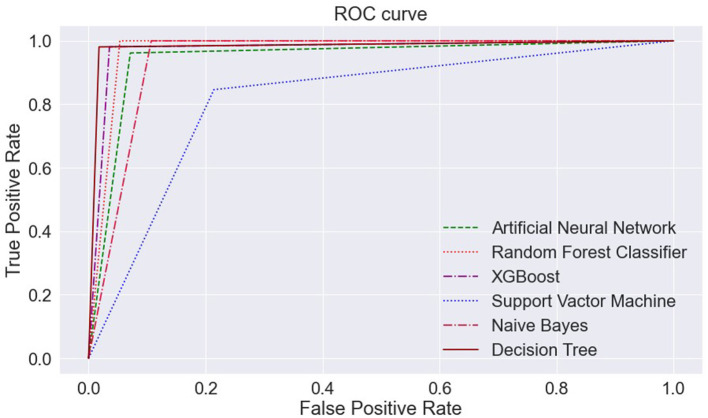
Counts of cardiac arrest in men/women.

Figure 9 shows counts of cardiac arrest prediction “1” and “0” in each gender, male and female. We conclude that 100 “men” are prone to cardiac arrest, and around 75 “men” are risk free of cardiac arrest. Similarly, more than 200 “women” are prone to cardiac arrest, and ~140 “women” are risk free from cardiac arrest.

**Figure 10:**
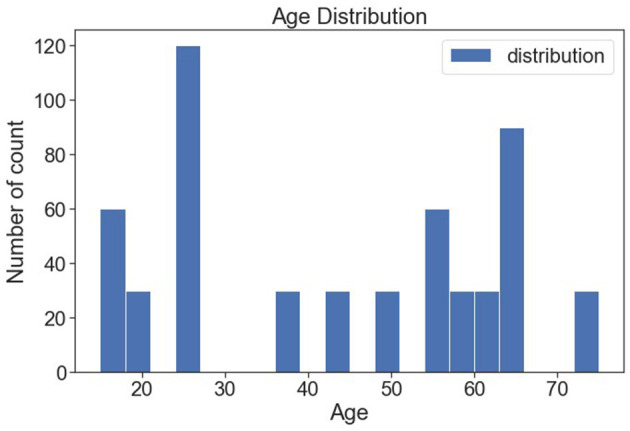
Age-wise distribution.

Figure 10 shows the various age distributions. It is observed from the bar plot that 25 years of people are seen more following 17 and 63 years of people are found more.

**Figure 11:**
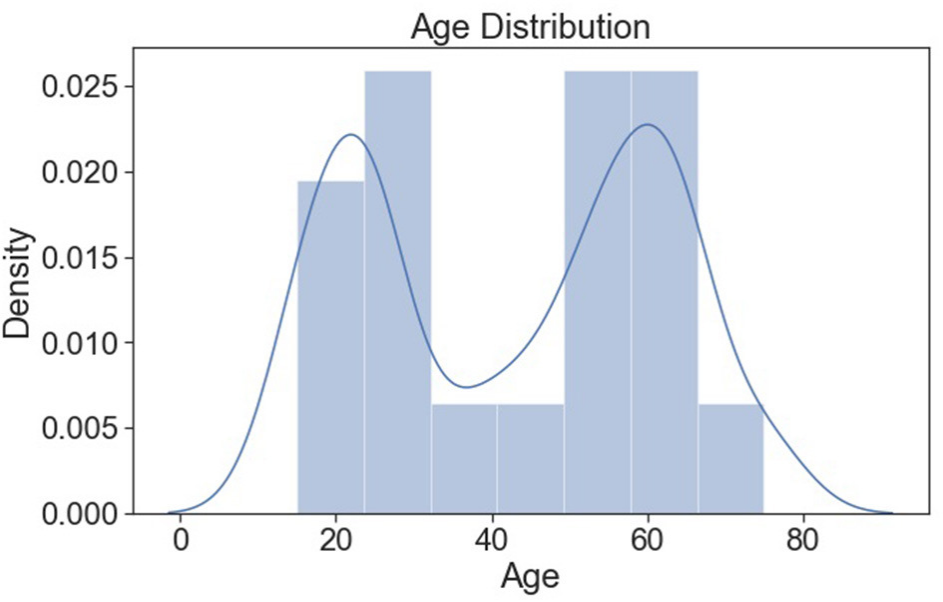
Age distribution (normalized).

Figure 11 shows the distribution of age, and it seems to be normally distributed.

## Implementation Details

### Experimental Setup

In this research, we implemented six supervised ML algorithms to evaluate the possibility of cardiac arrest and the performance of a classifier in a classification problem. For evaluation, the real-time dataset was collected using sensors and equipment as described in section Methodology. The dataset consists of 10 attributes, among which five main attributes, i.e., the vital signs of the patient, are mainly considered. For experimental execution, the “Data Streamer” tool was used in this study to collect the pulse rate every 2 min. Data Streamer is an open-source tool used to collect live data supporting capturing, analyzing, and visualizing real-time sensor data in Excel.

As described in section Methodology, the next step uses an ML model: ANN, RFC, XGBoost, SVM, NB, and DT classifier. Before applying the model to our collected data, the collected dataset was imported using “read_csv(),” and then the data was pre-processed as discussed in section Methodology. In the next step, we did feature selection using “StandardScaler()” where the vital signs of a patient were majorly considered. Furthermore, the dataset was split up into 80% training and 20% testing set. Then, models were applied to these data to check model accuracy. In the next phase, to test the performance of the classifier, various experimental metrics were computed. The contribution part of our research involves gender-wise analysis of risk/survival probability using the Kaplan-Meier algorithm, and age-wise risk probability for 10 years was formulated using the “XLSTAT” add-in tool in Excel. XLSTAT is an open-source software used to analyze, customize, and display results in Excel. Hence, the experiments discussed above have been carried out using Anaconda software in a Jupyter notebook python environment using ML libraries on an Intel(R) Core (TM) i5-3320M CPU @ 2.60 GHz system.

### Evaluation Metrics

The evaluation metrics of the model are formulated with the confusion matrix. For this, the calculation of values is measured based on:

- True positive (TP) = number of occurrences that are correctly determined.
- False negative (FN) = number of occurrences that are incorrectly predicted and not required.
- False positive (FP) = number of occurrences that are incorrectly predicted.
- True negative (TN) = number of occurrences that are correctly predicted and not required.

Based on this parameter, we have calculated four measurements with the given formula:

- Accuracy = *TN* + *TP*/*TN* + *TP* + *FN* + *FP*
- Precision = *TP*/*TP* + *FP*
- Sensitivity (Recall) = *TP*/*TP* + *FN*
- F1 Score = 2*(Precision*Recall/*Precision* + *Recall*)

These measurements help to know the cardiac risk associated with heart diseases.

### Results

**Figure 12:**
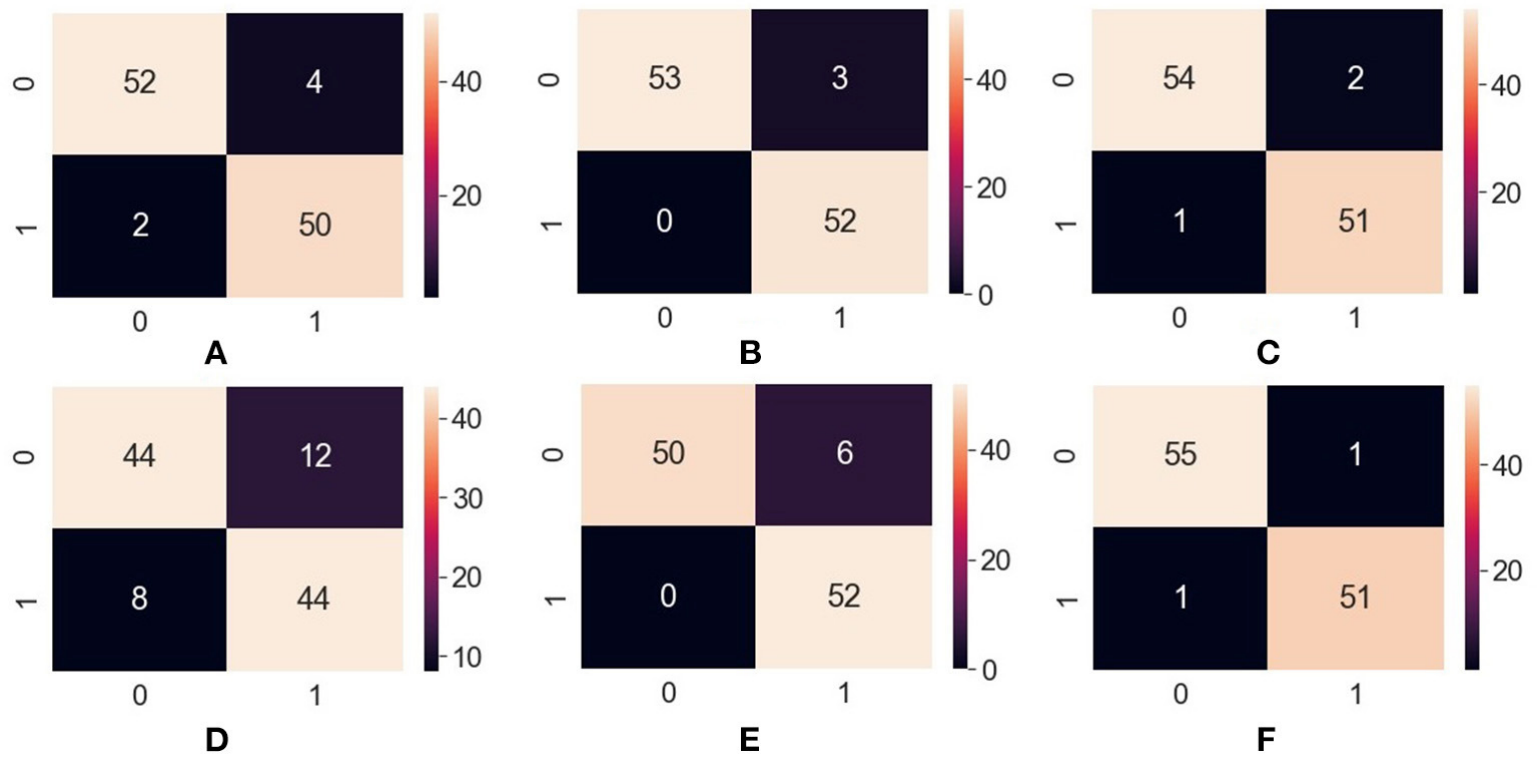
Confusion matrix—**(A)** ANN, **(B)** RFC, **(C)** XGBoost, **(D)** SVM, **(E)** Naïve Bayes, **(F)** Decision Tree. ANN, Artificial Neural Network; RFC, Random Forest Classifier; XGBoost, Extreme Gradient Boosting; SVM, Support Vector Machine.

After executing the ML model on the collected dataset, we found the accuracy, sensitivity, precision, and F1-score of each algorithm, as shown in Table 14. These measurements were calculated with the support of confusion matrix value TP, TN, FP, and FN for each algorithm shown in Figures 12A–F.

Table 17 shows the Precision, Recall, F1- score, and overall accuracy of ML algorithms. The value for Precision, Recall, and F1- score is calculated against 2—the class problem of having cardiac arrest risk and no cardiac arrest risk. The label “0” has been used for no risk in cardiac diseases, and “1” has been used for risk in cardiac disease.

**Table 17:** Model performance.

**Algorithm**	**Overall accuracy**	**Label**	**Precision**	**Recall**	**F1-score**
Decision tree	0.98	0	0.98	0.98	0.98
		1	0.98	0.98	0.98
Gradient boosting	0.97	0	0.94	0.98	0.97
		1	0.98	0.96	0.97
Random forest classifier	0.97	0	0.94	1.00	0.97
		1	1.00	0.94	0.97
Artificial neural network	0.94	0	0.92	0.96	0.94
		1	0.96	0.92	0.94
Support vector machine	0.81	0	0.78	0.84	0.81
		1	0.84	0.78	0.81
Naïve bayes	0.94	0	0.89	1.00	0.94
		1	1.00	0.89	0.94

Hence, Table 17 concludes that the DT outperforms all ML algorithms achieving overall 96% accuracy.

**Figure 13:**
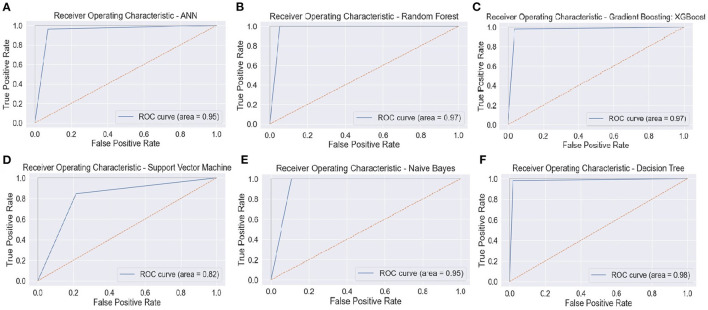
ROC curve—**(A)** ANN, **(B)** RFC, **(C)** XGBoost, **(D)** SVM, **(E)** Naïve Bayes, and **(F)** Decision Tree. ANN, Artificial Neural Network; RFC, Random Forest Classifier; XGBoost, Extreme Gradient Boosting; SVM, Support Vector Machine.

Figures 13A–F portrays the area under the curve (AUC) and receiver operating characteristic (ROC) curve. ROC curve depicts the graphical appearance to measure the model performance. It shows the true negative, and true positive values plotted at distinct levels of the threshold.

**Figure 14:**
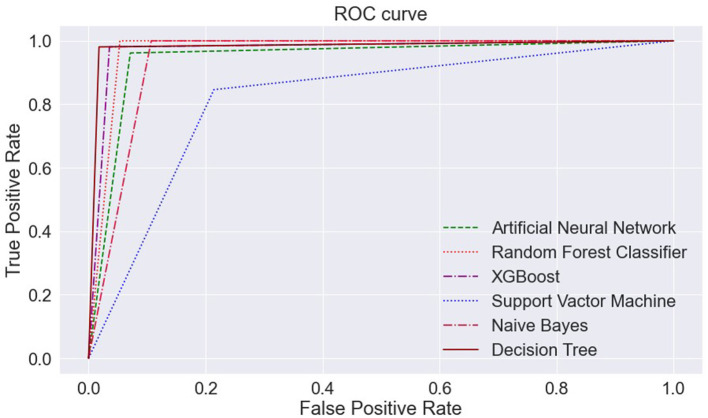
ML models—ROC curve. ML, machine learning; ROC, receiver operating characteristic.

The AUC curve represents the aggregate measures of performance at different threshold values. It also describes the probability value from the classification model to get an accurate result. If the AUC curve value shows 0 value, it predicts wrong results, and if 1, it means an exact model. Here, in Figure 14, the accuracy of all three classification models reaches 1 value, which means our prediction accuracy is highly accurate.

## Discussion, Conclusion, and Future Work

### Discussion

This study is aimed to detect and predict whether the patient is prone to cardiac arrest or not. This research was carried out using six ML classification algorithms, namely, ANN, RFC, XGBoost, SVM, NB, and DT classifier on a real-time collected dataset. Different tools were conducted for analyzing and classifying data on Intel(R) Core (TM) i5-3320M CPU @ 2.60 GHz system.

Dataset was split up into 0.8% training and 0.2% testing set, and then data pre-processing was done. To get the result, ML algorithms were applied to datasets to get the accuracy of the result. The results—accuracy, Precision, Recall, and F1-score—were generated using Python programming on Jupyter notebook.

**Figure 15:**
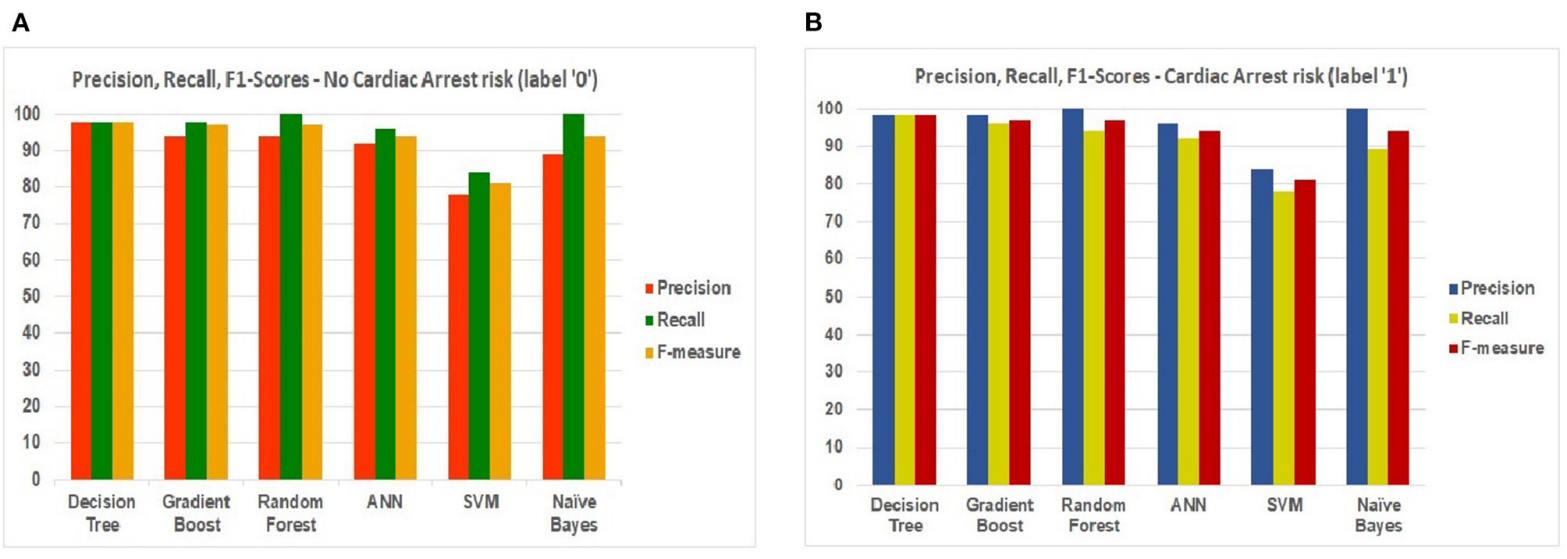
ML models—Precision, Recall, and F1-scores—for **(A)** label “0” **(B)** label “1.”

Figures 15A,B show the scores. Precision, Recall, and F1-scores of the patients having no risk or chances of cardiac arrest and patients with a risk of cardiac arrest. These scores help us to understand the measure of relevance of accuracy.

Comparing both charts, we conclude that “DT” reaches the maximum accuracy based on the scores retrieved from Precision, Recall, and F1 scores.

Furthermore, the main contribution of our research work shows an effective and efficient method for the prediction of cardiac arrest risk in gender-wise and different age-wise people in terms of survival probability. The method Kaplan-Meier shows statistical analysis in gender for showing the chances of survival probability. The result obtained by applying the Kaplan-Meier model to our collected dataset shows that female survival probability is more than that in men, as shown in Figure 6. Another method, Cox regression analysis, is another statistical analysis method that shows prediction/survival probability for the next 10 years of risk in cardiac arrest for different age groups. We can use this model to see cardiac risk in an individual. In addition, this method helps identify whether a person is at risk in the coming next 10 years using the score sheet displayed above.

### Conclusion

This study contributes classification techniques for detecting and comparing gender-based and age-based probability for cardiac arrest survival. The main objective of our work is to recognize cardiac arrest in a patient as early as possible using an ML model. Apart from that, our main contribution shows survival probability in an individual with gender-based and age-based using two effective methods: Kaplan-Meier and Cox regression methods. From the result, in detecting cardiac arrest risk in gender-based survival probability, female patients of our collected record show higher chances of survival than male patients. While in detecting cardiac arrest risk in age-based survival probability, patients with age 30 and more may have chances of cardiac risk as per our proposed model. From our classification model, the predicting results of the DT outperform the other ML classifiers.

### Future Study

This study can be further extended by adding more diseases and making predictions using different classifier models. In addition to that, we can add more features, and predictions can be made on a larger dataset.

## Data Availability Statement

The raw data supporting the conclusions of this article will be made available by the authors, without undue reservation.

## Author Contributions

SP and SA: conceptualization and design. SP, SA, and AS: data collection, manuscript writing, conceptualization, and design. SP, SA, AS, and KK: manuscript proofreading, manuscript writing, conceptualization, and design. SR: revisions, conceptualization, manuscript writing, and manuscript proofreading. All authors contributed to the article and approved the submitted version.

## Conflict of Interest

The authors declare that the research was conducted in the absence of any commercial or financial relationships that could be construed as a potential conflict of interest.

## Publisher's Note

All claims expressed in this article are solely those of the authors and do not necessarily represent those of their affiliated organizations, or those of the publisher, the editors and the reviewers. Any product that may be evaluated in this article, or claim that may be made by its manufacturer, is not guaranteed or endorsed by the publisher.
